# Relationships between the intention to use guidelines, behaviour of insurance physicians and their determinants

**DOI:** 10.1186/1472-6963-13-400

**Published:** 2013-10-09

**Authors:** Antonius JM Schellart, Feico Zwerver, Johannes R Anema, Allard J van der Beek

**Affiliations:** 1 Department of Public and Occupational Health, EMGO Institute for Health and Care Research, VU University Medical Center, Amsterdam, the Netherlands; 2 Research Center for Insurance Medicine AMC-UMCG-UWV, VU University Medical Center, Amsterdam, the Netherlands

**Keywords:** Insurance physician, Guidelines for depression, Intention, Behaviour, ASE model

## Abstract

**Background:**

We studied the intention of a group of insurance physicians to use the guidelines for depression, and their behaviour in disability assessments. We considered attitude, social norm and self-efficacy, knowledge/skills and stimuli, based on the Attitude - Social norm - self-Efficacy model (ASE model) as possible determinants of both intention and behaviour.

The aim of this study was to understand the determinants of insurance physicians’ behaviour when they are expected to use guidelines in daily practice.

**Method:**

A representative sample of 42 insurance physicians participated in this study. Cross-sectional data were collected by means of a questionnaire based on the ASE model. We developed the questionnaire on the basis of literature and ascertained the content validity of it. Behaviour was made to comprise both “use of the guidelines” and “change in disability assessment behaviour” by the insurance physicians. Reliability analyses were performed to form additive scales of the ASE constructs. These scales were analysed with structural equations modelling (LISREL), by modifying a start model into a final model with a good fit, within theoretical constraints. In these analyses special attention was paid to the fact that the sample size was small.

**Results:**

The most important determinants of the intention and the self-reported use of the guidelines, were: the influence of colleagues, the self-efficacy of the insurance physicians in their use of the guidelines, and the way the guidelines were implemented. The intention to use the guidelines for depression was not associated with the self-reported use of these guidelines, but there proved to be a faint, positive association with the self-reported change in assessment behaviour.

**Conclusions:**

Almost all the insurance physicians in this study intended to use at least elements of the guidelines. Their intention, self reported use of the guidelines and self-reported change in assessment behaviour were explored with help of the ASE model. The model suggested relationships between intention, self reported use of the guidelines and self-reported change in assessment behaviour on the on the one hand and various determinants on the other hand. Be that as it may, we see opportunities to improve insurance physicians’ guideline adherence by offering them a multifaceted training in which they learn to apply the guidelines for depression.

## Background

Since the introduction of evidence-based guidelines in health care the adherence of physicians to those guidelines has been subject to research [1-3]. The implementation of guidelines and the adoption of guidelines by physicians in daily practice appeared to be a complex process, much of which is still unknown [4]. Researchers have used various theories and methods to explore the adherence of physicians to guidelines [4-6]. Researchers have often used the Theory of Planned Behaviour (TPB) and its derivative, the Attitude, Social norm, self-Efficacy model (ASE model) [7,8], to investigate the behavioural aspects of the use of guidelines by physicians [9-13]. The ASE model and its precursor TPB are relevant models for studying intention and behaviour. For instance, Armitage and Conner [14] found in a meta-analysis of TPB studies that the model accounted for around 39% of the variance in intention and 27% of the variance in behaviour. The aim of the present study is to explore and understand the physicians’ behaviour towards guidelines with help of the ASE model.

**Figure 1:**
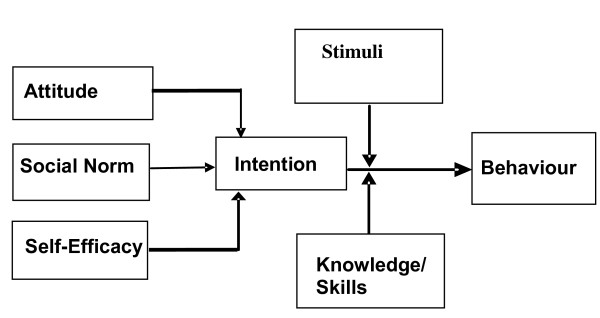
**ASE-model **[8]**.**

The ASE model explains behaviour by linking attitude, social norm and self-efficacy with behavioural intention and actual behaviour [15]. In addition to the three determinants of behavioural intention and actual behaviour, factors such as ‘knowledge and skills’ and ‘stimuli’ may play a role. The immediate precursor of behaviour is intention, but in order to predict whether a physician intends to use guidelines we need to know the physician’s attitude towards the guidelines. In the ASE model, intention is also determined by social-influence and self-efficacy. An individual physician may feel pressured by colleagues or a staff physician to use guidelines. The degree of self-efficacy that a physician feels when applying guidelines can also determine his intention to use guidelines. According to the ASE model, the link between the intention and the actual use of the guidelines may be stronger when the use of guidelines is promoted by facilitating factors, such as a multifaceted implementation strategy, or when guidelines are easily accessible. This link may be weakened by barriers (negative stimuli) between intention and use, e.g., a lack of practical applicability, a lack of agreement about the relevance of the guidelines, and a lack of supporting staff [6]. The ASE model is presented in Figure 1.

Although researchers have succeeded in identifying barriers preventing the use of guidelines, and have recommended improvements in implementation strategies, the adherence level of physicians to guidelines often remains low [1,16-18]. Therefore, we need to increase our insight into the entire process, from the dissemination of guidelines to their actual use by physicians in daily practice.

Both the TPB and the ASE model have been used to explain, among other things, the behaviour of physicians and patients concerning guidelines in an occupational health context [12,19,20]. In this study we concentrate on guideline adherence in the field of insurance medicine. Insurance physicians (IPs) working in the Netherlands have recently been confronted with guidelines for the first time. An IP is a physician with a registration in insurance medicine, acquired after four years of post-graduate education. The IP assesses disability claims by employees by doing an interview and an examination, and by filling in a functional ability list, all recorded in a medical work disability report [19]. Insurance medicine guidelines cover the work disability assessment of an employee. This assessment starts with a diagnosis that takes co-morbidity into account. It is followed by the physician’s view on the severity of the disorder and the risk factors affecting the client’s general well-being. The assessment includes the physician’s view on a possible treatment or therapy, or other interventions. The physician finally sums up the client’s current limitations and offers a prognosis regarding the development of the disorder [20]. Recently, validated ASE constructs were used successfully in order to explain the intention of insurance physicians to communicate with clients in disability assessments [21], and the professional intention and behaviour of insurance physicians concerning the process and content of disability assessments of clients [22,23].

We decided to concentrate on the guidelines for depression because on a global scale depression is on the increase as a major cause of long-term disability [24,25]. For the assessment of the diagnosis ‘depression’ the guidelines refer to the Diagnostic Statistic Manual IV.

We used the ASE model as a systemic framework for the identification of behavioural markers predicting the intention and the self-reported use of the guidelines, and the various stimuli that might influence the IPs’ behaviour regarding their use of the guidelines. In this model we hypothesized positive relations between *intention* and self-reported *use* of the guidelines, and subsequently between self-reported use of the guidelines and self-reported change of assessment behaviour resulting from application of the guidelines.

The research questions are, firstly: Which are the most important determinants of a) the intention to use the guidelines for depression, b) the self-reported use of these guidelines, and c) the self-reported change in their assessment behaviour? Secondly: Is there a positive relationship between the intention to use the guidelines for depression and not only the self-reported use of these guidelines but also the self-reported change in assessment behaviour? And finally: Is there a positive relationship between the self-reported use of the guidelines for depression and the self-reported change in assessment behaviour?

## Method

The Medical Ethical Committee of VU University Medical Center approved the study. We developed a questionnaire with the ASE model as its theoretical basis [8], focusing on the application of the guidelines for depression by IPs. The questions were derived from research literature on guideline adherence and focused on the ASE model. The questionnaire included 14 theory-based constructs from the ASE model, such as intentions, attitudes, social norm, self-efficacy, knowledge/skills, and stimuli in relation to guidelines in general and to the guidelines for depression in particular. Most questions were rated on a 5-point Likert scale, ranging from ‘totally disagree’ to ‘totally agree’, while some were rated on a 10-point rating scale. As most questions found in the literature could not be used for the purpose of our study, we adapted them to the context of IPs. In the questionnaire each set of questions concerning an ASE concept was preceded by an introduction text in order to frame the context of these questions. The questionnaire also contained questions about the background of the IPs.

The questionnaire was tested for content validity (comprehensiveness and relevance). In the first phase of this pilot study two IPs completed the questionnaire thinking out loud, which enabled us to check whether the questions were interpreted correctly. After some adjustments to this first version, 15 IPs filled in the second version, and gave their comments afterwards. These comments were taken into account for the final version of the questionnaire. The constructs of the ASE model used in the questionnaire, the number of items for each construct, and the background of the IPs are summarized in Table 1. The items of each ASE-construct in the questionnaire with the references to the specific literature used can be found in the Additional file 1.

**Table 1:** Constructs of the ASE model, and the background of the insurance physicians

**Constructs of the ASE model**	**#**
Attitude to the use of guidelines in general	9
Attitude to the specific use of the GD	9
Social influence of colleagues on the use of the GD	9
Social influence of important others in the use of the GD	5
Self-efficacy concerning the use of the GD	11
Knowledge and skills concerning the use of the GD	8
Stimuli affecting the use of the GD concerning structure and layout	3
Stimuli affecting the use of the GD concerning the implementation	3
Stimuli affecting the use of the GD concerning organizational factors	9
Stimuli affecting the use of the GD concerning the tools delivered	16
Stimuli affecting the use of the GD concerning the quality	11
Intention to use the GD	10
Use of the GD	4
Change in assessment behaviour due to the GD	3
**Background of IPs**	
Age in years	1
Number of working hours per week	1
Number of clients with depression assessed per month	1
Assessment time for depressed clients	1
Years working as physician	1
Years of working as insurance physician	1
Intensity of kind of professional activities	6
Statutory background of the assessments of (the majority of) clients	5
Industrial insurance boards the Insurance physicians had worked with before	1
Gender	1
Registered as insurance physician	1
Employed of the Institute	1

*GD* = Guidelines for Depression.

*Institute* = the Dutch Institute for Employee Benefits Schemes.

# = Number of items in the questionnaire.

The guidelines for depression were distributed and implemented at the Dutch Institute for Employee Benefits Schemes (Institute) in 2007 without any specific training. From the autumn of 2008 till March 2009 insurance physicians employed by the Institute were invited to follow a four-day postgraduate course in applying the guidelines to the disability assessment of clients with depression. Our aim was a minimum of 40 and a maximum of 50 participants. We used various strategies when recruiting IPs for our study: oral presentations, an e-mail to all IPs (roughly 900), and an ad on the restricted, employee-only website of the Institute. Furthermore, the post-graduate course was accredited as an incentive. Thanks to all these efforts we managed to tempt 42 IPs into participating in our study. Due to this recruitment procedure it could be that the participating IPs were more interested in the guidelines for depression than their colleagues who declined our invitation. The inclusion criteria were: The participant must be officially registered as an IP or currently following the post-academic Insurance Medicine colloquium, and he or she must be making disability assessments on a regular basis.

The forty-two participating IPs were asked to fill in the questionnaire at the start of the training, before the collection of any other data. Their answers were used to determine which constructs from the questionnaire were suitable for further analysis. A reliability analysis, including an item-analysis, was performed for the 14 constructs of items that were theoretically assumed to form an additive scale. We considered a Cronbach’s alpha of 0.65 as the minimum of internal consistency of a scale. These reliability analyses were performed in the SPSS 15.0 program.

We recoded items on the questionnaire concerning the background of the IPs into 15 background variables. To select the possibly relevant background variables we used the Ordinary Least Squares regression backward selection (Pin = 0.05, Pout = 0.10) option of the SPSS 15.0 program, with all background variables as independent variables and each of the 14 scale variables as a dependent variable. We included eight background variables that had a meaningful association with one or more scale variables in further analyses in order to take possible confounding effects into account. The correlations between the 14 scale variables and the 8 background variables were calculated in Prelis 2.72 [26]. To interpret the relationships between the variables we used Lisrel 8.72 [27]. This enabled us to examine the correlation matrix in a structural equations model with observed variables, i.e. a path model.

As the size of our study sample was smaller than the number of parameters, the parameter estimates were unreliable with N = 42. In such a case it is difficult to determine how one can change the model to produce a good model fit. We decided to artificially increase the number of participants to N = 200, which can be considered as a optimum number for the sample size in structural equations models [28]. This does not influence the estimated direct effects but decreases the standard errors of these effects and thereby enhances the significance of the estimated effects. Furthermore, the model fit decreases and, assuming the same degrees of freedom, as a consequence the model has a greater chance to be rejected. In our view it is this last aspect that particularly justifies an artificial increase of the number of participants to the optimum number if one wants to explore relationships in a path model.

Following the theoretical ASE model we formulated a structural start model with the scale variables as endogenous variables, and the background variables as exogenous variables. Subsequently, with an estimated start model containing 11 endogenous scale variables and six exogenous background variables, we fitted the model. This means we closed non-significant parameters between endogenous variables and opening parameters with significant modification indices (>3.84) within the theory-based constraints. We then checked whether the model fit was good [29]. This required the (Normal Theory Weighted Least Squares) Chi-square of the model to be small, i.e. less than twice the number of degrees of freedom (df). It required the Root Mean Square Error of Approximation (RMSEA) and the Standardized Root Mean Square Residual (SRMR) to be less than 0.05. It also required the Comparative Fit Index (CFI) to be equal to or greater than 0.90. Furthermore, we verified whether the Q-plot of the standardized residuals crossed the diagonal for normal distribution, and whether the correlation of estimates was less than 0.7.

To verify the estimated coefficients of the fitted model for N = 42, we deleted the direct effects of the exogenous variables on the endogenous variables, resulting in an estimated ASE model with relationships between the endogenous variables only. We estimated this model for N = 200 to look at confounding effects of the exogenous variables and the model fit. In addition, we estimated this model for N = 42, in which the number of parameters was smaller than the sample size of N = 42.

## Results

### Background variables

The self-reported background variables of the IPs are presented in Table 2. We checked whether the 42 IPs who participated in this study were a representative sample of the total group of IPs employed by the Institute (n = 900) with respect to gender, age and working hours per week. In the *group of participants* the mean age was 51 years (SD = 14.90) (CI = [46.5; 55.7]), 47.8% was female, and the physicians worked 31.68 hours per week on average (SD = 9.31) (CI = [28.9; 34.5]). The mean age of the *total group* was 49 years, 41.7% female, working 32 hours per week on average (distribution measures of the total group could not be calculated) [22]. The mean age and the average number of hours worked by an IP of the total group were within the 95% CI of the participants’ group average. With regards to other important aspects (i.e. number of years’ working experience as an insurance physician, being registered as such) the group of participants closely resembled a representative sample of 231 IPs from the same population, mentioned in the study of Steenbeek et al. in 2008 [22]. This made us conclude that we were in fact working with a representative sample.

**Table 2:** Background variables of insurance physicians (n = 42)

**Description of the background variables**	**%**
**Age in years **^§^* (mean = 51. 10; sd = 6.34)	
50 years and younger = 1	45.2
Older than 50 years = 2	54.8
**Number of working hours per week **^**§**^ (mean = 31.69; sd = 9.31)	
Part-time (≤ 34 hours) = 1	47.6
Full-time (> 34 hours) = 2	52.4
**Number of clients with depression per month **^**§**^ (mean = 7.02 clients; sd = 4.98)	
0 - 1 client = 1	9.5
2 - 4 clients = 2	19.0
5 - 8 clients = 3	38.1
9 - 10 clients = 4	21.4
11 or more clients = 5	11.9
**Assessment time for depressed clients **^§^* (mean = 144.5 minutes; sd = 54.41)	
30 - 45 minutes = 1	4.8
46 - 110 minutes = 2	19.0
111 - 180 minutes = 3	57.1
181 - 210 minutes = 4	14.3
211 - 240 minutes = 5	4.8
**Years working as physician **^§^* (mean = 22.67 years; sd = 5.65)	
10 - 18 years = 1	23.8
19 - 27 years = 2	52.4
28 - 31 year = 3	23.8
**Years working as insurance physician **^§^* (mean = 15.38 years; sd = 7.79)	
6 - 9 years = 1	26.2
10 - 22 years = 2	50.0
23 - 31 years = 3	23.8
**Intensity of kind of professional activities **^§^* (mean = 3.95 activities; sd = 0.79)	
1-3 activities = 1	23.8
4 activities = 2	52.4
5 activities = 3	23.8
**Gender**	
Male =1	52.4
Female = 2	47.8
Registered as insurance physician *	
Yes = 1	85.7
No = 2	14.3
Employee of the Institute *	
Yes = 1	78.6
No = 2	21.4

^§^ Recoded into an ordinal variable.

* Not used in the structural equations model.

Institute – Dutch Institute for Employee Benefits Schemes.

On average the physicians assessed 7 clients with a depression (SD 5.0) per month. Nearly all of them reported that they intended to use, or continued to use, certain elements from the guidelines for depression. Approximately 50% reported that they used the complete guidelines for depression, and approximately 85% reported that they used at least some elements of the guidelines for depression. Approximately 50% of the IPs reported that the use of these guidelines affected their assessment behaviour.

### ASE scale variables

The 14 ASE scale variables for the 42 IPs are presented in Table 3. The average Cronbach’s alpha of the scales was 0.79.

**Table 3:** ASE scale variables

**Name of ASE scale variables**	**Description**	**#**	**Theoretical (empirical)**		**Median**	**Mean**	**SD**	**α**
			**Min**	**Max**				
Attitude *	Attitude to the use of the guidelines in general	9	9 (18)	45 (44)	32.00	31.50	6.09	0.76
Attitude GD	Attitude to the specific use of the GD	9	9 (19)	45 (44)	33.00	33.14	5.33	0.77
Social Norm Colleagues	Social influence of colleagues on the use of the GD	9	9 (15)	29 (29)	22.50	22.74	3.67	0.69
Social Norm Others	Social influence of important others in the use of the GD	5	5 (5)	25 (22)	16.00	15.38	4.36	0.81
Self-Efficacy	Self-efficacy concerning the use of the GD	11	11 (24)	55 (48)	36.00	35.31	5.38	0.75
Knowledge & Skills	Knowledge and skills concerning the use of the GD	8	8 (16)	40 (39)	28.00	27.81	5.34	0.77
Format GD	Stimuli affecting the use of the GD concerning structure and layout	3	3 (13)	30 (27)	20.00	19.98	3.11	0.90
Implementation ^§^	Stimuli affecting the use of the GD concerning the implementation	3	1 (1)	5 (5)	3.00	3.10	1.14	0.71
Institute *	Stimuli affecting the use of the GD concerning organisational factors	9	9 (10)	45 (38)	26.00	25.38	6.97	0.84
Tools ^§^*	Stimuli affecting the use of the GD concerning the tools delivered	16	1 (1)	5 (5)	3.00	2.98	1.14	0.89
Quality GD	Stimuli affecting the use of the GD concerning the quality	11	11 (24)	55 (51)	40.00	39.02	6.10	0.84
Intention	Intention to use the GD	10	10 (19)	50(46)	35.00	35.05	5.80	0.76
Use GD ^§^	Use of the GD	4	1 (1)	5 (5)	3.00	3.02	1.16	0.65
Change AB ^§^	Change in assessment behaviour due to the GD	3	1 (1)	3 (3)	2.00	1.98	0.60	0.86

Scores of insurance physicians (n = 42), with the number of items (#), the minimum of the scales (Min), the maximum of the scales (Max), median of the scores, mean of the scores, the standard deviations (SD), and the reliability of the scales (Cronbach’s alpha; α). GD = Guidelines for Depression. Institute = Dutch Institute for Employee Benefits Schemes.

^§^ Recoded into an ordinal variable.

* Not used in the structural equations model.

# Number of items in the questionnaire.

### Structural equations

**Figure 2:**
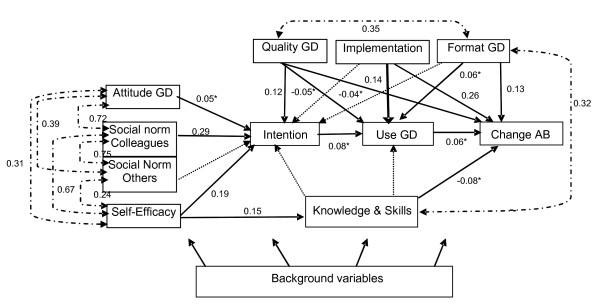
**Start model Legenda: GD = Guideline Depression, AB = Assesment Behaviour.** Model fit parameters: Chi-square 91.732, df = 73, p = 0.07; RMSEA = 0.036; SRMR = 0.067, CFI = 0.974. Straight lines indicate direct effects (beta matrix), double-arrowed dotted lines indicate associations in the disturbance terms (psi matrix); single arrowed dotted lines indicate direct effects which had to be included in the model to get positive definite matrices. All coefficients p < 0.05; except (*), p is not significant, at N = 200, artificially. Explained variance (R^2^) of endogenous variables: Attitude to the GD (0.10); Social Norm, influence of colleagues on acceptation of the GD (0.05); Social Norm, influence of important others in adherence to the GD (0.10); Self-efficacy the use of the GD (0.14); Knowledge and skills concerning the GD (0.10); Stimulus in the use of the GD due to the format of the guideline (0.05); Stimulus in the use of the GD due to the implementation of the guidelines (0.14); Stimulus in the use of the GD by the quality of the guideline (0.09); Intention to use the GD (0.24); Use of the GD (0.12); Change in assessment behaviour due to the GD (0.25).

The relationships in the start model between the endogenous variables (beta and psi matrix) are presented in Figure 2, together with the model fit parameters. These parameters indicated that the start model did not fit well (SRMR = 0.067; modification indices > 3.84). Direct effects of six background variables on endogenous variables in the model (not shown here) were significant: gender, number of clients with depression assessed by an IP per month, number of working hours per week by an IP, IP’s employment record and two different types of legislation regarding workers’ disability.

**Figure 3:**
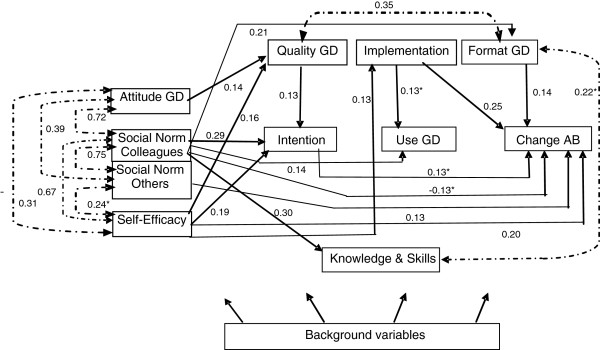
**Final Model Legenda: GD = Guideline Depression; AB = Assessment Behaviour.** Model fit parameters at N = 200, artificially: Chi Square 45.438, df = 72, p = 0.994; RMSEA = 0.0; SRMR = 0.0411, CFI = 1.000. All coefficients p < 0.05; except (*), p < 0.10, at N = 200, artificially. Explained variance (R^2^) of endogenous variables: Attitude to the GD (0.10); Social Norm, influence of colleagues on acceptation of the GD (0.05); Social Norm, influence of important others in adherence to the GD (0.10); Self-efficacy the use of the GD (0.14); Knowledge and skills concerning the GD (0.16); Stimulus in the use of the GD due to the format of the guideline (0.10); Stimulus in the use of the GD due to the implementation of the guidelines (0.16); Stimulus in the use of the GD by the quality of the guideline (0.14); Intention to use the GD (0.25); Use of the GD (0.14); Change in assessment behaviour due to the GD (0.30).

We adjusted the direct effect between the endogenous variables in the model in order to obtain good fit parameters. The final model, which contained the same direct effects of exogenous variables on the endogenous variables as the initial model, is presented in Figure 3, and shows the direct effects between the endogenous variables (beta matrix) and the associations between the (disturbance terms of) endogenous variables (psi matrix). The model fit parameters, and other parameters (Q-plot, modification indices, correlation of estimates), indicated that the model fit was good. The explained variance was highest for the intention to use the guidelines (0.25) and for the self-reported change in assessment behaviour (0.30).

**Figure 4:**
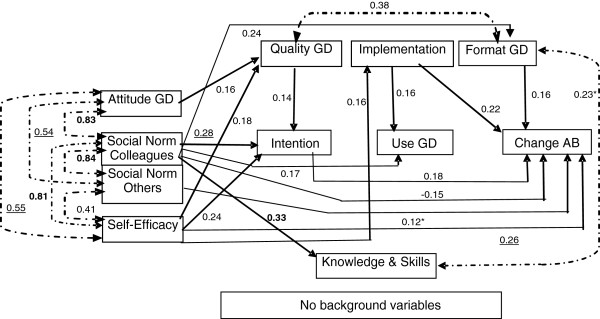
**Final Model of endogenous variables only Legenda: GD = Guideline Depression; AB = Assessment Behaviour.** 1) Model fit parameters at N = 200, artificially: Chi Square 26.058, df = 31, p = 0.719; RMSEA = 0.0; SRMR = 0.0499, CFI = 1.000. All coefficients p < 0.05; except (*), p < 0.10; at N = 200, artificially. Explained variance (R^2^) of endogenous variables: Knowledge and skills concerning the GD (0.11); Stimulus in the use of the GD due to the format of the guideline (0.06); Stimulus in the use of the GD due to the implementation of the guidelines (0.03); Stimulus in the use of the GD by the quality of the guideline (0.08); Intention to use the GD (0.23); Use of the GD (0.06); Change in assessment behaviour due to the GD (0.23). 2) Model fit parameters at N = 42: Chi Square 5.369, df = 31, p = 1.0; RMSEA = 0.0; SRMR = 0.0499, CFI = 1.000.Coefficients are the same as for the model with N = 200. All coefficients are not significant (p > 0.10), except bold coefficients (p < 0.05) and underlined coefficients (p < 0.10); at N = 42. Explained variance (R^2^) of endogenous variables are the same as for N = 200.

With N = 200 and N = 42 respectively, the same model for the endogenous variables only is presented in Figure 4, i.e. the direct effects of the exogenous variables on the endogenous variables were omitted from the model. The coefficients for direct effects between the endogenous variables (beta matrix) in Figure 4 largely resembled those in Figure 3, i.e. there are only small confounding effects of the exogenous variables. Most associations between the (disturbance terms of) endogenous variables (psi matrix) were a lot stronger in Figure 4 than in Figure 3. With N = 200, most coefficients were significant (p < 0.05), while with N = 42 most of them were not. The model fit parameters were good for both the model with N = 200 and the model with N = 42. With the estimated models presented in Figure 3 and Figure 4 we were able to answer the research questions.

Concerning the first research question, the most important determinants with regard to *the intention to use the guidelines* (a) were: The influence of colleagues on acceptance of the guidelines, self-efficacy and, to a lesser extent, the perceived quality of the guidelines. The most important determinants with regard to the *self-reported use of the guidelines* (b) were: The influence of colleagues regarding acceptance of the guidelines and, to a lesser extent, the facilitators for using the guidelines provided by the implementation strategy. The most important determinants with regard to *the self-reported change in assessment behaviour* (c) were: self-efficacy leading to the use of the guidelines, facilitators for using the guidelines provided by the implementation strategy and, to a lesser extent, the influence of colleagues and important others resulting in acceptance of the guidelines. In addition, self-reported change was somewhat stimulated by the lay-out of the guidelines.

With respect to the second and third research questions, the final model showed no direct relation between *intention* and the *self-reported use* of the guidelines. The answers indicated a faint, positive relationship between *intention to use* and *change in assessment behaviour*. We did not find a relationship between the *self-reported use* of the guidelines and *change in assessment behaviour*. Furthermore, we found some associations among the ASE determinants themselves.

## Discussion

Nearly all participating IPs reported that they intended to use certain elements of the guidelines for depression. In addition, approximately 50% of the IPs changed their assessment behaviour due to the guidelines. The influence of colleagues on acceptance of the guidelines, self-efficacy in using the guidelines, and facilitators for using the guidelines provided by the implementation strategy proved to be the most important determinants for the intention to use the guidelines. That we did not find a relationship between a change in assessment behaviour and the use of the guidelines - which we theoretically expected - can be attributed to IPs’ assumption that they already worked according to the guidelines. So why would they change their assessment behaviour? At the Institute IPs are kept informed on new developments by their senior IP. Not to our surprise we found that the IPs were influenced by the opinions of both their colleagues and their senior IP. Self-efficacy is a personal determinant for the intention to use the guidelines, and it can be enhanced by further education. We also found that the implementation strategy for the guidelines influences the self-reported use of the guidelines. Hence we think that guideline adherence can be strengthened by focusing on the implementation strategy.

The use of a theoretical psychological model to describe the behaviour of the IPs with regard to the use of guidelines is clearly a strong point of our study. The questionnaire we developed included all relevant constructs from the ASE model. Furthermore, the scales we formulated for these constructs had moderate to good reliability, which enabled us to analyze the relationships between the ASE constructs as *observed variables in a structural model*. The IPs participating in our study were a representative sample on the basis of important socio-demographic aspects.

The main limitation of this study is the cross-sectional design. Despite the use of Lisrel, which analyses associations of variables for determining cause and effect, we could draw no causal conclusions. Another limitation of our study is that we could not investigate validity aspects of the operationalised ASE constructs beforehand, other than the content validity. For example, the self-reported change in behaviour was not measured against data on the assessment behaviour at baseline. The results of this study should be interpreted with caution because of their self-reported nature, the limited number of participants, and the fact that we had to artificially increase the sample in order to modify the start model into a model with a good fit. Furthermore, although the IPs were a representative sample they *volunteered* for the course on how to apply the guidelines, therefore selection bias is possible (for example, the enthusiasm of participants was more than average). This bias may explain the fact that we found only a (weak) association between the intention of IPs and the change in their assessment behaviour, no association between their intention and their actual use of the guidelines depression, and no association between their use of the guidelines and the change of assessment behaviour. Thus, the estimated relations in the model could have been stronger if we had included IPs who did not participate the course as well.

In this study the self-reported use of the guidelines turned out to be rather high in comparison with reports from related fields, such as primary care, clinical care and occupational health care. After all, we found a self-reported use of the guidelines of 85%. This high percentage may (partly) be due to the fact that in insurance medicine physicians have a strong legal obligation to use guidelines in general. Former research among IPs in the Netherlands reported an adherence of 90% to protocols for semi-structured assessment interviews in disability assessments [30]. In primary care, the overall adherence to 70 guidelines within a period of 10 years was 67% [17], whereas another study reported a low adherence (39%) to the guidelines for mental health problems by occupational physicians [12]. In general, it was suggested that 30-40% of the patients do not receive care according to current scientific evidence [31] as recorded in guidelines. A review of 30 studies focusing on the attitude of clinicians towards guidelines reported a high satisfaction rate regarding clinical practice guidelines, but also concerns about the applicability of the guidelines, their contribution towards cost reduction, and their potential side-effect of more litigation [32].

To our knowledge, there is only one other study that has focused on the attitude of IPs towards guidelines [33]. However, this study was carried out in Belgium, where there are no specific insurance medicine guidelines. The study indicated a positive attitude of IPs towards clinical guidelines, but a limited use.

Most studies that used psychological theoretical models for describing the behaviour of physicians with regard to guidelines, such as the TPB or the ASE model, concluded that the model used could only partly explain the variations in the adherence of the physicians to these guidelines [11,18]. In our study we could only confirm this conclusion. However, as in other studies, the theoretical psychological model did prove to be useful for describing the behaviour of the IPs with regard to the use of the guidelines [5,11]. We now have indications as to which forces stimulate the use of the guidelines by IPs, and what stimuli might work. Unlike other studies that describe the behaviour of the physicians with the help of a theoretical psychological model [5,10-12,34,35], we used structural equation modelling, which provided more insight into the complex processes determining the behaviour of IPs, and changes in that behaviour when they are expected to apply guidelines in daily practice. The paths formed by the associations that we found between the variables and the determinants of the ASE model showed us how IPs’ intention and use of the guidelines can be influenced and improved.

Our study indicates that apparent peer influence on the use of the guidelines makes it feasible to monitor IP-behaviour towards guideline adherence with specific performance indicators, and to provide them with feedback. Although the rate of self-reported (intentional) use of the guidelines in our study was already high, improvements in guideline adherence can be achieved by increasing the self-efficacy of the IPs. We found that IPs with more self-efficacy were more willing to change their behaviour in order to apply guidelines in daily practice. In addition, given the positive association that we found between implementation and self-reported use of the guidelines and self-reported change in assessment behaviour, increased efforts to improve the implementation might result in an increase in IPs’ guideline adherence. That may be a good starting point for interventions towards increasing guideline adherence. The most important ASE determinants for the intention to use the guidelines for depression, the self-reported use of the guidelines by IPs, and the change in their assessment behaviour seem to be influenced by colleagues, by the self-efficacy of the IPs, and by various stimuli occurring when implementing the guidelines.

## Conclusions

Guideline adherence of insurance physicians was explored with help of the ASE model, showing a relationship between guideline adherence and various determinants without fully confirming the ASE model. The most important determinants for the intention to use guidelines and the self reported use of guidelines by insurance physicians and the change in their assessment behaviour were: the influence of colleagues, self-efficacy and the implementation of the guidelines. The intention to use the guidelines was associated with change in assessment behaviour, and 50% of the insurance physicians changed their assessment behaviour due to the implementation of the guidelines for depression. We see opportunities to improve insurance physicians’ guideline adherence by offering them a multifaceted training in which they learn to apply the guidelines for depression.

## Competing interests

The authors declare that they have no competing interests.

## Authors’ contributions

The authors declare that they participated in the study and made the following contributions to the study, and that they have seen and approved the final version. AJMS and FZ developed the questionnaire, performed the analyses and wrote the manuscript. AJMS, FZ, JRA, and AJvdB contributed to the conception and design of this study. JRA and AJvdB commented on the manuscript. AJMS, JRA and AJvdB will act as guarantors of this study. All authors read and approved the final manuscript.

## Pre-publication history

The pre-publication history for this paper can be accessed here:

http://www.biomedcentral.com/1472-6963/13/400/prepub

## Supplementary Material

Additional file 1 ASE constructs in questionnaire. Click here for file
