# Supplementary material for: Relationships between the intention to use guidelines, behaviour of insurance physicians and their determinants
Source: BMC Health Serv Res. 2013 Oct 9;13:400. doi: 10.1186/1472-6963-13-400 (PMC3851955; doi:10.1186/1472-6963-13-400)
Supplement: Additional file 1 — ASE constructs in questionnaire. [file 1472-6963-13-400-S1.doc]

**Additional file 1. ASE constructs in questionnaire.**

All items are scored with Likert Scale (1-5) 1 = strongly disagree, 5 = strongly agree, if not reported otherwise. See the reference list [A1-A16] at the end of this appendix.

**Attitude towards the use of guidelines in general [A1-A4]**

| 1. There are too many guidelines in insurance medicine practice as it is |
| --- |
| 2. The union of insurance physicians is responsible for guidelines |
| 3. The national Health Council is competent to develop guidelines |
| 4. Guidelines improve the quality of care |
| 5. Clients are too diverse for uniform guidelines to be used |
| 6. Guidelines are important for having insurance physicians work uniformly |
| 7. Guidelines lead to cookbook medicine |
| 8. Working according to guidelines leads to more efficiency in the assessments |
| 9. Guidelines increase insurance physicians’ work satisfaction |

**Attitude towards the specific use of the guidelines for depression [ A2, A5, A6]**

| 1. The guidelines for depression can help the IP make complex decisions |
| --- |
| 2. The guidelines for depression stimulate professionalism in IPs |
| 3. The guidelines for depression prove too rigid for applying them to individual clients |
| 4. The guidelines for depression enhance the quality of the assessment |
| 5. The guidelines for depression can improve the relationship between IP and client |
| 6. The guidelines for depression are a threat to the autonomy of the IP |
| 7. Applying the guidelines for depression prevents professionals from making themselves familiar with new insights concerning depression |
| 8. I agree with the contents of the guidelines for depression |
| 9. My attitude towards the guidelines for depression is positive |

**Social influence of colleagues on the use of the guidelines for depression [A1, A2, A7, A8]**

| 1. My fellow insurance physicians do not fully accept the the guidelines for depression |
| --- |
| 2. My fellow insurance physicians do not apply the guidelines for depression, so why should I ? |
| 3. If my staff physician would check our use of the guidelines for depression, I might apply the guidelines more often |
| 4. Do you know fellow insurance physicians who use the guidelines for depression? Yes, no. |
| 5. Has your opinion on the guidelines for depression been influenced by the experiences of  fellow insurance physicians? Yes, positively. Yes, negatively. No. |
| 6. Do you think that the guidelines for depression are inductive to more collaboration between professionals? Yes, no, not known. |
| 7. If Yes:  A. more collaboration between insurance physicians?  B. more collaboration between insurance physicians and occupational health physicians?  C. more collaboration between insurance physicians and the GP or specialist treating a client? |

**Social influence of others on IPs’ use of the guidelines for depression [A9, A10]**

| 1. My fellow insurance physicians at the front office think that I should apply the guidelines for depression |
| --- |
| 2. Other insurance physicians (not at my front office) think that I should apply the guidelines for depression |
| 3. My senior physician thinks that I should apply the guidelines for depression |
| 4. I think my clients expect me to apply the guidelines for depression |
| 5. I think clients appreciate my use of guidelines such as the guidelines for depression |

**Self-efficacy concerning the use of the guidelines for depression [A2, A8, A11, A12, A13]**

| 1. I feel sufficiently equipped for applying the guidelines for depression |
| --- |
| 2. The guidelines for depression have a positive effect on the quality of my assessments |
| 3. The guidelines for depression help me to: |
| A. Add structure to my assessment |
| B. Take away my doubts |
| C. Substantiate my decisions |
| D. Write down my findings in a sound disability report |
| E. Prepare the assessment interview |
| F. Freshen up my knowledge |
| 4. I find the information presented in the guidelines for depression:  Too complex; Just right; Too simple, or No opinion |
| 5. What is your verdict on the quality of the guidelines for depression?  A. The aim of the guidelines is: Unclear; Reasonably clear; or Fully clear  B. Assessing the prognosis to me is: Unclear; Reasonably clear; or Fully clear |

**Knowledge and skills concerning the use of the guidelines for depression [A9, A10]**

| 1. I have the knowledge one needs to apply the guidelines for depression |
| --- |
| 2. I have the skills to work in accordance with the guidelines for depression |
| 3. I feel the needs to further train and practice the use of the guidelines for depression |
| 4. I am able to organize my work in such a way that I can apply the guidelines for depression |
| 5. Learning to apply the guidelines for depression takes more time than I have at my disposal |
| 6. I have difficulties integrating the use of the guidelines for depression in my daily work routine |
| 7. Present (disability) legislation leaves enough room for working in accordance with the guidelines for depression |
| 8. I think applying the guidelines for depression is practically feasible |

**Stimuli affecting the use of the guidelines for depression concerning structure and layout [A14, A15]**

| 1. How would you score the layout of the guidelines for depression? Scale 1-10. 1 = very bad, 10 = very good. |
| --- |
| 2. Can you find the information that you need in the guidelines for depression? Scale 1-10 |
| 3. Can you recognize the relevant elements in the guidelines for depression which you need when assessing work disability? Scale 1-10 |

**Stimuli affecting the use of the guidelines for depression concerning the implementation [A2, A12, A14]**

| 1. I was sufficiently educated on the use of the guidelines for depression in 2007 |
| --- |
| 2. The implementation of the guidelines for depression in 2007 has been useful for my work on disability assessments. |
| 3. How satisfied are you with the 2007 training you received concerning the implementation of the guidelines for depression? Scale 1-10, 1 = very bad, 10 = very good |

**Stimuli affecting the use of the guidelines for depression concerning organizational factors [A5, A7, A11, A14]**

| 1. My use of the guidelines for depression is being stimulated by the following factors within the Institute: |
| --- |
| A. Management support |
| B. Good facilities |
| C. Considering the work load the guidelines have been implemented at the right time |
| D. Application of the guidelines saves time |
| E. The guidelines fit my work routine |
| F. The insurance medicine context of these guidelines is obvious |
| G. The guidelines do justice to actual assessment situations |
| 2. The Institute paid sufficiently attention to the guidelines for depression |
| 3. The guidelines for depression tie in with the other guidelines at the Institute |

**Stimuli affecting the use of the guidelines for depression concerning the tools delivered [A15]**

| 1. The tools provided have made the guidelines for depression easier to use in my daily work |
| --- |
| 2. The tools provided have helped me to fully understand the guidelines for depression |
| 3. My use of the guidelines for depression has been supported sufficiently by the tools provided |

**Stimuli affecting the use of the guidelines for depression concerning the quality [A1, A5, A7, A14]**

| 1. Application of the guidelines for depression enhances the quality of the assessments |
| --- |
| 2. The guidelines for depression represent a convincing source of advice |
| 3. The guidelines for depression are evidence base |
| 4. The contents of the guidelines for depression are scientifically valid |
| 5. The guidelines for depression were compiled with care |
| 6. The guidelines for depression result from an unbiased synthesis of expert opinions |
| 7. Clients´ perspectives and preferences were incorporated in the development of the guidelines for depression |
| 8. The information in the guidelines for depression is complete when it comes to: |
| A. the diagnostics of depression |
| B. the treatment of depression |
| C. the assessment of functional capacities |
| 9. The guidelines for depression fit in with the insurance physicians’ practice |

**Intention to use the guidelines for depression [A1, A2, A7, A9, A10, A16]**

| 1. I have the intention to use or keep using elements from the guidelines for depression |
| --- |
| 2. I expect to use elements from the guidelines for depression in the near future |
| 3. I intend to use or keep using the complete guidelines for depression |
| 4. I think the guidelines for depression are useful for taking decisions concerning the assessment of the work limitations |
| 5. Application of the guidelines for depression should be compulsory |
| 6. My clients benefit from the implementation of the guidelines for depression |
| 7. In my opinion there are clients to whom the guidelines for depression cannot be applied |
| 8. The guidelines for depression will probably be used in appeal cases |
| 9. The guidelines for depression will probably contribute to a decrease in lost appeal cases |
| 10.The guidelines for depression will contribute to more uniformity in the work disability assessments of clients with depression |

**Use of the guidelines for depression [A8, A10, A14]**

| 1. Do you have the guidelines for depression (on paper or in digital form)? Yes, No. |
| --- |
| 2. Have you read the guidelines for depression? Fully, Largely, Hardly, Not at all. |
| 3. Have you been using the guidelines for depression in practice? Yes, No. |
| 4. How many times did you consult the guidelines for depression? Never, 1-5 times, 6-10 times, over 10 times. |

**Changes in assessment behaviour due to the guidelines for depression [A8, A9, A14]**

| 1. My assessments have changed since I have started using the guidelines for depression. |
| --- |
| 2. Specific information from the guidelines for depression made me change my way of assessing work disability claims. |
| 3. In my opinion the assessment of clients with depression has changed at the Institute since the implementation of the guidelines for depression. |

The Institute – Dutch Institute for Employee Benefits Schemes

**References Appendix**

[A1] Heidrich J, Behrens T, Raspe F, Keil U**: Knowledge and perception of guidelines and secondary prevention of coronary heart disease among general practitioners and internist. Results from a physician survey in Germany**. *Eur J Cardiovasc Prev Rehabi.* 2005;**12**:521-529

[A2] Leemrijse CJ, Plas GM, Hofhuis H, van de Ende CH: **Compliance with the guidelines for acute ankle sprain for physiotherapists is moderate in the Netherlands: an observational study.** *Aust J Physiother* 2006;**52**:293-299

[A3] Bochud M, Cornuz J, Vader JP, Kamm W, Burnand B: **Are internist in a non prescriptive setting favourable to guidelines? A survey in a Department of Internal Medicine in Switzerland.** *Swiss Med Wkly* 2002;**132**:201-206

[A4] Tunis SR, Hayward RSA, Wilson MC, Rubin WR, Bass EB: **Internist’ Attitudes about Clinical Practice Guidelines***. Ann Intern Med* 1994;**120**:956-963

[A5] Formoso G, Liberati A, Magrini N: **Practice guidelines: useful and “participative” method? Survey of Italian physicians by professional setting.** *Arch Intern Med* 2001;**161**:2037-2042

[A6] Huis in ’t Veld MHA, Voerman GE, Hermens HJ, Vollenbroek-Hutten MMR: **The receptiveness toward remotely supported myofeedback treatment.** *Telemedicine and e-Health* 2007;**13**:293-300

[A7] Haagen EC, Nelen WLDM, Hermens RPMG, Braat DDM, Grol RPTM, Kremer JAM: **Barriers to physician adherence to a subfertility guideline.** *Hum Reprod* 2005 Dec;**20**(12):3301-6

[A8] Formoso G, Moja L, Nonino F, Dri P, Addis A, Martini M, Liberati A: **Clinical Evidence: a useful tool for promoting evidence-based practice?** *BMC Health Services Research* 2003;**3**:24

[A9] Rebergen D, Hoenen J, Heinemans A, Bruinvels D, Bakker A, van Mechelen W: **Adherence to mental health guidelines by Dutch occupational physicians.** *Occup Med (Lond)* 2006 Oct;**56**(7):461-8

[A10] Heinemans A, Rebergen D, Bakker A, Hoenen J: **Vragenlijst Sociaal-medische begeleiding en Werkbeleving bedrijfsartsen.** Bureau Richtlijnen NVAB, Utrecht 2001 (in Dutch)

[A11] Echlin PS, Upshur RE, Markova TP: **Lack of chart reminder effectiveness on family medicine resident JNC-VI and NCEP III guideline knowledge and attitudes.** *BMC Fam Pract* 2004 Jul 5;**5**:14

[A12] Backwood B, Wilson-Barnett J: **The impact of nurse-directed protocolised-weaning from mechanical ventilation on nursing practice: a quasi-experimental study.** *International Journal of Nursing Studies* 2007;**44**:209-226

[A13] Van der Steen JT, Ooms ME, Ribbe MW, van der Wal G: **Decisions to treat or not to treat pneumonia in demented psychogeriatric nursing home patients: evaluation of a guideline.** *Alzheimer Disease an Associated Disorders* 2001;**15**:119-128

[A14] Koek A. **Eindrapportage ‘Implementatie van het Regionaal Decubitusprotocol in gooi zuid’ Vragenlijst voor de evaluatie*.*** Stichting Transmurale Zorg Gooi en Vechtstreek, Hilversum 2005 (in Dutch)

[A15] The AGREE Collaboration: **Appraisal of Guidelines for Research & Evaluation (AGREE) Instrument.** St George’s Hospital Medical School, London 2001

[A16] Asano TK, Toma D, Stern HS, McLeod RS: **Current awareness in Canada of clinical practice guidelines for colorectal cancer screening.** *Canadian Journal Surg* 2004;**47**:104-108
